# Analysis Tool Web Services from the EMBL-EBI

**DOI:** 10.1093/nar/gkt376

**Published:** 2013-05-11

**Authors:** Hamish McWilliam, Weizhong Li, Mahmut Uludag, Silvano Squizzato, Young Mi Park, Nicola Buso, Andrew Peter Cowley, Rodrigo Lopez

**Affiliations:** EMBL Outstation–European Bioinformatics Institute, Wellcome Trust Genome Campus, Hinxton, CB10 1SD Cambridge, UK

## Abstract

Since 2004 the European Bioinformatics Institute (EMBL-EBI) has provided access to a wide range of databases and analysis tools via Web Services interfaces. This comprises services to search across the databases available from the EMBL-EBI and to explore the network of cross-references present in the data (e.g. EB-eye), services to retrieve entry data in various data formats and to access the data in specific fields (e.g. dbfetch), and analysis tool services, for example, sequence similarity search (e.g. FASTA and NCBI BLAST), multiple sequence alignment (e.g. Clustal Omega and MUSCLE), pairwise sequence alignment and protein functional analysis (e.g. InterProScan and Phobius). The REST/SOAP Web Services (http://www.ebi.ac.uk/Tools/webservices/) interfaces to these databases and tools allow their integration into other tools, applications, web sites, pipeline processes and analytical workflows. To get users started using the Web Services, sample clients are provided covering a range of programming languages and popular Web Service tool kits, and a brief guide to Web Services technologies, including a set of tutorials, is available for those wishing to learn more and develop their own clients. Users of the Web Services are informed of improvements and updates via a range of methods.

## INTRODUCTION

The European Bioinformatics Institute (EMBL-EBI) provides access to a wide range of biological data resources and bioinformatics applications (1). As well as being available via a web browser, many of these services provide Web Services interfaces based on REST (Representational State Transfer) (2) or SOAP (Simple Object Access Protocol—http://www.w3.org/TR/soap). At present, the BioCatalogue (3), a registry of biological Web Services, lists 724 Web Services provided by EMBL-EBI. The availability of Web Services interfaces allows the integration of data and analysis tools into other tools, applications, web sites, pipeline processes and analytical workflows, while avoiding the need to maintain the databases and programs locally.

Using Web Services, functionality from many databases and analysis tools from a wide range of service providers can be combined to create complex analytical workflows and mashups. This can add value to existing database search or analysis tool results by incorporating data or analysis results from other services. In this article, we describe the current Web Services from the EMBL-EBI for data search, entry retrieval, analysis tools and their use together.

## WEB SERVICES

### Data search and retrieval

Individual data resources, such as ChEBI (4), ENA (5), Gene Expression Atlas (6) and UniProt (7), provide web interfaces and Web Services (Table 1) tailored to the specifics of their data and the usage patterns required by consumers of their data. These interfaces cater well for searches against the specific resource and address searches and data retrieval operations concerning data from the specific resource; however, this can prove to be an issue when access to multiple data sources is required because each data resource has to be handled separately.

**Table 1.** Data resource-specific Web Services

Topic	Web Services
Genomes	Ensembl BioMart, Ensembl Genomes REST API
Nucleotide sequences	ENA Browser
Protein sequences	PRIDE BioMart, UniProt.org, UniProt BioMart
Small molecules	ChEBI WS, PSICQIC (ChEMBL)
Gene expression	ArrayExpress, Gene Expression Atlas API
Molecular interactions	PSICQIC (IntAct)
Reactions, pathways and diseases	BioModels, PSICQIC (Reactome), Rhea
Protein families	InterPro BioMart
Literature	Europe PMC Web Service
Ontologies	Ontology Lookup Service (OLS), QuickGO, SBO::Web Services, WSMIRIAM

For cases where a more general approach is required, for example, searching across several different databases using one query, the EB-eye (8) Web Service is a possible solution. In addition to searching across the multiple databases available at the EMBL-EBI, the EB-eye Web Service allows navigation through the cross-references network, formed by data entries referencing other entries the same database or in other databases. The EB-eye Web Service can return information held within specified fields of the data entries, and allows searches to be refined by domain or using Boolean logic.

The EB-eye Web Service is limited to retrieving data from selected fields stored in its indexes, so it is complemented by the dbfetch and WSDbfetch (9) services, which provide single or batch whole entry data retrieval based on entry identifiers. The entry data are often available in a range of data formats, for example, UniProtKB entries are available for retrieval in UniProtKB flat-file format, fasta sequence format, GFF (http://gmod.org/wiki/GFF3), UniProt XML format, UniProt RDF/XML format and SeqXML (http://seqxml.org/0.4/seqxml_doc_v0.4.html).

### Analysis tool services

As well as the data search and retrieval services, a range of analysis tool services are also available (Table 2), including sequence similarity search [e.g. FASTA (10) and NCBI BLAST (11)], multiple sequence alignment [e.g. Clustal Omega (12) and MUSCLE (13)], pairwise sequence alignment, protein functional analysis [e.g. InterProScan (14) and Phobius (15)], etc. Most of the analysis tool services are implemented using a job dispatcher framework, JDispatcher (16), which provides a web interface and SOAP & REST Web Services interfaces. The interfaces of these Web Services are largely consistent, easing the learning curve and aiding re-use of supporting services.

**Table 2.** Analysis tool and general data web services

Topic	Web Services
Data retrieval	Dbfetch^a^, WSDbfetch^a^
Identifier mapping	PICR^a^, UniProt.org ID Mapping^a^
Multi-database search	EB-eye^a^
Multiple sequence alignment	Clustal Omega, ClustalW2, DbClustal, Kalign, MAFFT, MUSCLE, MView, PRANK, T-Coffee
Pairwise sequence alignment	Lalign, EMBOSS tools: matcher, needle, stretcher and water, and the Wise2 tools: GeneWise, PromoterWise and Wise2DBA
Phylogeny	ClustalW2 Phylogeny
Protein functional analysis	InterProScan, Phobius, RADAR
Sequence format conversion	EMBOSS seqret, MView, Readseq
Sequence operations	CENSOR, Seqcksum
Sequence similarity search	FASTA, FASTM, NCBI BLAST, PSI-BLAST, PSI-Search, WU-BLAST
Sequence statistics	SAPS and the EMBOSS tools: pepinfo, pepstats and pepwindow
Sequence translation	EMBOSS tools: backtransambig, backtranseq, sixpack and transeq
Structure analysis	DaliLite, MaxSprout
Text mining	Whatizit^a^

^a^Services not implemented using JDispatcher framework.

## COMBINING SERVICES

### Search and data retrieval

**Figure 1.**
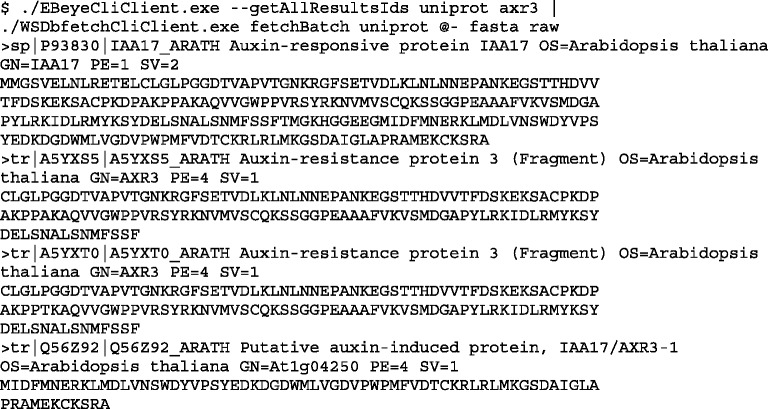
Combining the EB-eye and WSDbfetch Web Services to perform a search in UniProtKB for the term ‘axr3' and retrieve the corresponding entries in fasta sequence format using the sample .NET clients provided for these services.

The EB-eye and the dbfetch or WSDbfetch Web Services provide a modular approach for performing a database search and retrieving required data in a specified data format. The sample command-line clients provided for these services are capable of being chained together in such a way that these processes can be combined in a single command (Figure 1). For more complex queries or data-retrieval requirements, it may be necessary to use either the sample clients within a script with additional commands to handle any required data transformations, or a client specific to those Web Services, which implements additional logic.

A similar process can be used to combine a sequence similarity search (e.g. FASTA or NCBI BLAST) with data retrieval to automatically pull back the set of hit sequences found for a search. The download functionality provided in the web interface for sequence similarity search results on the EMBL-EBI web site uses this process to obtain the sequences from the dbfetch service and return them to the user.

More advanced workflows and data pipeline processes can be built by combining further analysis tool services. For example, the result from a sequence similarity search can be directly used as input for a multiple sequence alignment, needing only the job identifier to be passed between services in the cases of MView (17) and DbClustal (18).

### Web Services in other services

Because Web Services are provided as functionally distinct modules they are well suited for use as components inside web interfaces, where they can provide additional functionality. Some examples are given below.

#### Sequence similarity search

The sequence similarity search Web Services are used in a number of web sites: the Ensembl Genomes BLAST (19) is provided using the WU-BLAST (20) Web Service; the PDBe ‘Sequence Search’ (http://www.ebi.ac.uk/pdbe/?tab=home&subtab=sequencesearch) uses the FASTA Web Service; and the UniProt.org ‘Blast’ (http://www.uniprot.org/blast/) uses the NCBI BLAST Web Service.

#### Text search

The EB-eye Web Service is used to provide the text search functionality for other services at the EMBL-EBI. This is often combined with a query filtering process, which recognizes the input of entry identifiers that the underlying resource can handle, and passes other search terms to the EB-eye Web Service to retrieve a relevant set of identifiers for further processing. The ENA Text Search (http://www.ebi.ac.uk/ena/) uses this process. In Ensembl Genomes, the EB-eye Web Service is used to provide a search facility across the many genome databases in the collection.

#### Multiple sequence alignment

The ‘Align’ section (http://www.uniprot.org/align/) of the UniProt.org web site uses the Clustal Omega Web Service to handle the alignment of the input sequences. The webPRANK (21) web interface (http://www.ebi.ac.uk/goldman-srv/webprank/) to the PRANK multiple sequence alignment tool uses the PRANK Web Service provided by JDispatcher to run the analysis.

These Web Services are also being used in desktop applications and as components of workflows implemented within other tools, for example, Blast2GO (http://www.blast2go.com/), BlastStation (http://www.blaststation.com/), Bioclipse (22) and T-COFFEE (23). Additionally published workflow definitions, which consume these services, created using Taverna (24) and other workflow design tools can be found in repositories such as myExperiment (25).

## DISCUSSION

The availability of Web Services from the EMBL-EBI allows developers to integrate additional functionality into their programs and web sites without having to worry about maintaining their own copies of the required databases or software involved, or indeed the resources for the storage and execution of the databases and software. This addresses requirements for such functionality, while minimizing the duplication of effort. Reflecting the existing use of bioinformatics tools in general, the modular nature of the Web Services allows users to combine services to create powerful data pipeline and analytical workflows.

At the EMBL-EBI we are seeing the volume and proportion of Web Services traffic continuing to increase. During 2011 the analysis tool services at EMBL-EBI processed ∼36 million analysis jobs, of which ∼30 million were submitted via the SOAP/REST Web Services interfaces. For 2012 this rose to 50 million jobs of which ∼43 million were submitted via the SOAP/REST Web Services interfaces. Regular web browser traffic now comprises <50% of the total web traffic. While much of this change is likely due to the adoption of Web Services in some genome annotation pipelines, this also reflects the usage of Web Services to provide modular functionality as part of applications and in other web sites. Apart from the maintenance of the existing public Web Services, we continue to develop new Web Services and integrate significant data workflows.

We are mindful that Web Services are often used as part of complex pipelines or highly automated processes. These situations present additional requirements regarding the maintenance of interface consistency, etc. Changes to Web Services interfaces and locations can break third-party applications that have grown up around them, and as noted in Schultheis *et al.* (26), there is a range of Web Service persistency and availability from different institutions. At the EMBL-EBI we aim to meet our users’ needs for persistency and availability, and we use a range of communication channels (e.g. mailing lists, news feeds and Twitter) to keep users informed of service changes and availability (see http://www.ebi.ac.uk/Tools/webservices/help/faq).

To aid in the use of our Web Services, we provide significant documentation (e.g. method descriptions, FAQ, tutorials) and sample clients in a range of programming languages (e.g. C#, Java, Perl, PHP, Python, Ruby and VB .NET). During 2013, we plan to further engage with our users to provide further examples of potential data flow combinations in the online documentation. We also provide training courses and helpdesk support for the use of specific Web Services and general help with the development of clients for Web Services available from the EMBL-EBI.

## FUNDING

Funding for open access charge: European Molecular Biology Laboratory (EMBL).

*Conflict of interest statement.* None declared.
